# The Isotropic and Cubic Material Designs. Recovery of the Underlying Microstructures Appearing in the Least Compliant Continuum Bodies

**DOI:** 10.3390/ma10101137

**Published:** 2017-09-26

**Authors:** Sławomir Czarnecki, Tomasz Łukasiak, Tomasz Lewiński

**Affiliations:** Department of Structural Mechanics and Computer Aided Engineering, Faculty of Civil Engineering, The Institute of Building Engineering, Warsaw University of Technology, 00-637 Warsaw, Poland; t.lukasiak@il.pw.edu.pl (T.Ł.); t.lewinski@il.pw.edu.pl (T.L.)

**Keywords:** auxetic materials, topology optimization, free material design, compliance minimization, homogenization, isotropic and cubic composites

## Abstract

The paper discusses the problem of manufacturability of the minimum compliance designs of the structural elements made of two kinds of inhomogeneous materials: the isotropic and cubic. In both the cases the unit cost of the design is assumed as equal to the trace of the Hooke tensor. The Isotropic Material Design (IMD) delivers the optimal distribution of the bulk and shear moduli within the design domain. The Cubic Material Design (CMD) leads to the optimal material orientation and optimal distribution of the invariant moduli in the body made of the material of cubic symmetry. The present paper proves that the varying underlying microstructures (i.e., the representative volume elements (RVE) constructed of one or two isotropic materials) corresponding to the optimal designs constructed by IMD and CMD methods can be recovered by matching the values of the optimal moduli with the values of the effective moduli of the RVE computed by the theory of homogenization. The CMD method leads to a larger set of results, i.e., the set of pairs of optimal moduli. Moreover, special attention is focused on proper recovery of the microstructures in the auxetic sub-domains of the optimal designs.

## 1. Introduction

Available nowadays, the additive manufacturing techniques can be used to produce materials of a slowly varying inhomogeneity with very diverse and rare mechanical properties and with almost arbitrary complex micro/nanostructure. An interesting overview of the recent developments in the area of the so-called extremal- or meta-materials (elastic and non-elastic) can be found in [1,2]. The papers on material design are mainly aimed at modeling and manufacturing of composites of extreme effective moduli, while just the computer modeling plays an important role in the design of new materials. The composites resulting from mixing two or more isotropic materials with different volume fractions or resulting from mixing one isotropic material with the voids of different size and shape to form new porous materials are the subject of the most rapidly developed methods and techniques for modeling the meta-materials. The term *mixing* is here understood as the multiscale modeling and homogenization-based approach used in analytical derivation of explicit formulae for the effective moduli of higher rank laminates and in calculations of their values. The question of fundamental importance for the development of modeling and manufacturing of composites is: which elasticity tensors are realizable? The answer to this question is now known and is very promising from the point of view of the prospects for the composites manufacturing technology, since, as Milton and Cherkaev noted (see page 117 in [3]): “…any given positive fourth order tensor satisfying the usual symmetries of elasticity can be realized as the effective elasticity tensor of a two-phase composite comprised of a sufficiently compliant isotropic phase and a sufficiently rigid phase configured in an suitable microstructure”. This realization can be performed by a properly designed lamination of very compliant and very stiff isotropic phases. However, the microstructure of the so constructed Hooke tensor is usually not unique and very often difficult to fabricate.

A very specific class of meta-materials (not necessarily isotropic and elastic) are auxetic materials—the most widely investigated extremal-materials with a negative Poisson’s ratio (see [4,5,6,7,8,9,10,11]), and the most important challenge nowadays is to develop a methodology of reconstructing microstructures of auxetic materials in such a way that their elastic properties correspond exactly to the design properties reflecting their specific properties such as synclastic behavior, high resistance to indentation, shear loads and high toughness. These and other properties that distinguish auxetic materials from other materials are the subject of many studies and numerical analyses aimed at their mass production in a wide variety of fields (see, e.g., [12,13,14,15,16,17,18,19,20,21,22,23,24,25,26,27,28]). Currently, at least three methods of developing meta-materials (including auxetic materials) dominate.

In the first approach, applying pressure, heating, external load to non-auxetic composites (e.g., compacting and forming a solid mass of material by heat or pressure without melting it to the point of liquefaction—sintering process) or direct laser writing (DLW) technique result in the proper modifications of the microstructure of negative Poisson’s ratio (see, e.g., [24,25,29,30]). Forming thermodynamically stable auxetic phases on the base of properties exhibited by the molecules; modifying solid structure by introduction of the auxetic layers and nanochannels; and investigating the role of molecular shape and its influence on macroscopic elastic properties are the basic directions of research developed in the second approach (see, e.g., [31,32,33,34,35,36,37]). In the third approach, a wide variety of techniques, methods and models is proposed in both the design and manufacture of auxetic materials. The choice of a technique is generally a question of scale: from macroscopic down to the molecular level (see, e.g., Figure 3, page 385 in [6]). Revealing relationships between macroscopic behavior and the nature of the physical phenomena is essential in the design or predicting the physical properties of the meta-materials at the micro level (see, e.g., [38]). Expanding the analysis of such structures, in which the auxetic properties are observed, from micro- to macro-scale has become possible “…considering not only the base materials, but the internal structure and deformation mechanism…” (see [5]). Gradient honeycomb composite structures with hexagonal configurations, chiral or reentrant structures with or without inclusions, rotating rigidly or semi-rigidly with elastic, elastic-plastic, small or large deformations using buckling behavior, single- or double-array structures, perforated models, sheet-, anti-tetrachiral- or cross-like missing rib models and many others models are now the subject of an intense research (see [7,8,9,10,16,17,18,20,21,22,23,39,40,41]). Moreover, the topology and shape optimization, very often implemented jointly with the homogenization techniques, deserve particular attention (see [8,11,26,28,41,42,43,44,45,46]). Just the latter methods will be used in the present paper.

This paper is an extension of the previous works of the present authors (see [47,48,49,50,51,52,53]). The main objective of the paper is to find the distribution of bulk *k*(x) and shear *μ*(x) moduli (x ∈ Ω, Ω being the two-dimensional design domain) which minimizes the overall compliance of the structure. The unit cost of the design is assumed as the trace of Hooke’s tensor. The problem thus formulated, called Isotropic Material Design (IMD), is a constrained version of the Free Material Design (FMD), in which Kelvin moduli λ*_K_*(x) and eigenstate tensors **ω***_K_*(x) (*K* = 1, 2, 3 for two dimensional bodies—in short 2D) are design variables (see [49,50,54]). An extremely important feature of the non-homogeneous solutions, found by IMD method, is the emergence of subdomains where the Poisson ratio is negative (practically in the whole range from 0 to −1). The numerical results shown in the present paper for the deep beam supported at two bottom corner nodes and loaded with one vertical force applied to the upper face fully confirm this feature and clearly shows the auxetic subdomains. 

The IMD method delivers the optimal inhomogeneous distribution of the bulk and shear moduli. These inhomogeneous fields of the moduli may be interpreted as the homogenized fields of a composite whose representative volume element (RVE) is formed of two isotropic phases (numbered by 0 and 1) of given bulk and shear moduli (*k*_0_, *μ*_0_) and (*k*_1_, *μ*_1_), respectively. The inhomogeneity is modeled by varying geometry of the layout of these phases within RVEs corresponding to the subsequent points x, thus making RVEs x-dependent. The homogenized fields *k*^H^(x) and *μ*^H^(x) are constructed by the strict formulae of the theory of homogenization. The modeling includes the case of the phase 0 being a void, leading to the porous material solution.

The optimal layout of the phases within the RVEs is constructed by minimizing both the differences between *k*(x) and *k^H^*(x) as well as *μ*(x) and *μ^H^*(x) for subsequent points x ∈ Ω. Thus, calculated in this way the effective moduli *k^H^*(x) and *μ^H^*(x) correspond to an internal structure on the micro-scale, shown explicitly in the figures. Particularly interesting is the form and shape of the microstructures at those points where optimal material exhibits auxetic properties. The recovery of microstructures is performed by a direct combinatorial search. Alternatively, one can apply the available inverse homogenization techniques (see [55]).

The CMD method constructs the optimal inhomogeneous distribution of the moduli *a*, *b*, *c* of a cubic material (see [53]). According to [53] the optimal modulus *b* vanishes. By using combinatorial search a family of RVEs is constructed whose effective moduli exhibit the desired extremal properties. The elaborated RVEs make it possible to construct the optimal microstructure of the two optimum designs of a deep beam by using the IMD and CMD methods.

The 2D settings of the IMD and CMD methods deliver the isotropic and cubic optimal designs in two dimensions. By extension of these designs in the transverse dimension, we arrive at the 3D designs of the material of the underlying transverse isotropic and cubic microstructures, both being manufacturable, contrary to 3D isotropy, which cannot be easily constructed. The IMD and CMD methods have their strong and weak points. The IMD method leads to the design of given bulk and shear moduli; they are usually positive, except for some regions where one of them can vanish. Thus, in general, the design is non-degenerated. On the other hand, the optimal cubic material is always degenerated, since one of the moduli vanishes in the whole design domain, which is the characteristic feature of the single load problem (see [53]). The optimal cubic RVEs are geometrically instable, having zero stiffness due to shear. On the other hand, the cubic RVEs are much easier to manufacture; the design space is much bigger. Indeed, majority of the microstructures of the modern metamaterials are cubic (see, e.g., [56]). Having in view the mentioned virtues and vices of IMD and CMD, both the methods are discussed.

## 2. Isotropic and Cubic Material Design. Least Compliant 2D Structures

Let us start by recalling the basic notions of elasticity theory [57], referring also to large deformations. Let Ω ⊂ R^3^ be the set representing the undeformed body and Ω*^φ^* ⊂ R^3^, Ω*^φ^* = *φ*(Ω) be the set representing the body after it is deformed, where *φ*: Ω→R^3^ is the smooth enough, injective and orientation-preserving vector field defining the deformation. The sets Ω and Ω*^φ^* are called the *reference* and *deformed configurations*, respectively. Materials for which the Cauchy stress tensor **σ**(x*^φ^*) at any point x*^φ^* = *φ*(x) is completely determined by the *deformation gradient* ∇ *φ*(x) at the corresponding point x ∈ Ω (i.e., by Fréchet derivative of the mapping *φ* at x) are called *elastic* materials. A little more mathematically precisely: a material is elastic if there exists a mapping $C:\Omega \times M_{+}^{3}\to S^{3}$, called the *response function*, such that σ(φ(x))=C(x,∇φ(x)); here $M_{+}^{3},\;S^{3}$ denote the set of all real square matrices with positive determinant and the set of all symmetric matrices of 3rd order, respectively. A material is called *homogeneous in a reference configuration* Ω if its response function is independent of the point x ∈ Ω, i.e., σ(φ(x))=C(∇φ(x)), otherwise the material is called *non-homogeneous in a reference configuration* Ω. The term *isotropy* is in the simplest way interpreted as the property of material whose response to a given load is the same in all directions. A little more mathematically precisely, an elastic material is *isotropic at a point* x ∈ Ω if the response function satisfies the following condition: $\forall F\in M_{+}^{3}\;\forall Q\in O_{+}^{3}\;C\left(x,F\;Q\right)=C\left(x,F\right)$, i.e., if the Cauchy stress tensor is unaltered when the reference configuration is subjected to an arbitrary rotation $Q\in O_{+}^{3}$ around the point x ∈ Ω. If the above condition is met only for rotations Q∈G, where $G\subset O_{+}^{3}$ is the subgroup of the group $O_{+}^{3}$, then material is said to be *anisotropic at a point* x ∈ Ω. An elastic material is *isotropic* if it is isotropic at all points x ∈ Ω.

In the case of a linearized displacement-traction problem, the simplest mathematical formula on the response function for anisotropic elastic materials is reduced to the linear relation between the stress tensor **σ**(x) and symmetric part of the *displacement gradient* called *linearized strain tensor* **ε**(x), i.e., σ(x)=C(x,ε(x)), where C(x,ε(x))=C(x)ε(x) and $C=C\left(x\right)=C_{ij}{}_{kl}\left(x\right)e_{i}⊗e_{j}⊗e_{k}⊗e_{l}$, (*i*, *j*, *k*, *l* = 1, 2, 3) is the 4th rank Hooke tensor; the orthonormal vectors **e***_i_*, *i* = 1, 2, 3 span the Euclidean space of points in R^3^, the summation convention over small Latin indices being applied. The components C*_ijkl_* are subject to the symmetry conditions: *C_ijkl_* = *C_klij_*, *C_ijkl_* = *C_jikl_*, *C_ijkl_* = *C_ijlk_*, and to the positivity condition: *C_ijkl_ ε_ij_ ε_kl_* ≥ *cε_ij_ ε_j_* i.e., in Ω for some *c* > 0. Other properties of linear elastic materials known as isotropic-, cubic-, transversely isotropic-, tetragonal- hexagonal-, rhombic-, monoclinic-, and triclinic systems are defined mathematically by various spectral representations of elasticity tensor **C**, commonly known as: isotropic and orientation Hooke tensors [58]. The spectral decomposition formula involves independent constitutive moduli (eigenvalues and tensor products of eigenstates—often called projectors), whose number varies between 2 and 21 for isotropic and anisotropic Hooke tensors, respectively.

The present paper deals with the problem of the compliance minimization of a structure subjected to a traction load $t=\left(t_{1},t_{2}\right)$ applied to the given part Γ_1_ of the boundary Γ of the given, two-dimensional design domain Ω ⊂ R^2^. The body is fixed on the boundary Γ_2_, a part of Γ. The paper deals with two problems: (i) designing moduli of an isotropic body; and (ii) designing moduli of a body made of a cubic material. In the former optimum design problem the design variables are: the bulk *k* = *k*(*x*) and the shear moduli *μ* = *μ*(*x*), *x* ∈ Ω defining the non-homogeneous, isotropic, 4th rank Hooke tensors **C**:

(1) $$C\left(x\right)=2k\left(x\right)\;\Lambda_{1}+2\mu \left(x\right)\;\Lambda_{2}\;,\;x\in \Omega ,$$

where the projectors are

(2) $$\Lambda_{1}=\frac{1}{2}\delta_{ij}\delta_{kl}e_{i}⊗e_{j}⊗e_{k}⊗e_{l}\;,\;\Lambda_{2}=II-\Lambda_{1},$$

and **II** stands for the 4th rank identity tensor in 2D case. The representation of the flexibility tensor, or the tensor inverse to **C**, reads

(3) $$C^{-1}=\frac{1}{2k}\Lambda_{1}+\frac{1}{2\mu }\Lambda_{2}$$

The design variables are subject to the cost (or an isoperimetric) condition:

(4) $$\int_{\Omega }\mathrm{tr}\;C\;dx=\Lambda ,\;\Lambda =E_{0}\left|\Omega \right|=const,$$

where *E*_0_ is the referential elastic modulus, |Ω| represents the area of the design domain and tr C=2k+4μ. Condition (4) determines the set ℜ of admissible pairs (k,μ) of non-negative moduli corresponding to the isotropic Hooke tensor **C**.

The compliance is defined as the work of the given loading **t** done on the displacements $u=\left(u_{1},u_{2}\right)$ caused by the same loading. More precisely, let us define the linear form

(5) $$\forall v\in V\left(\Omega \right)\;f\left(v\right)=\int_{\Gamma_{1}}t\cdot v\;ds,$$

where $v=\left(v_{1},v_{2}\right)$ is a virtual, kinematically admissible field, or the field satisfying the condition: **v** = **0** on the boundary Γ_2_ (dot “·” denotes the scalar product **t**·**v** = *t_i_*·*v_i_*). The compliance of the structure is defined as the functional

(6) ℘: ℜ→R, ℘(k, μ) = f(u),

where the displacement field **u** depends on **C** and thus depends on (*k*, *μ*), which will be written as u=u(k,μ). Let us introduce the set

(7) $$\Sigma \left(\Omega \right)=\left\{\tau \in E_{s}^{2}|\forall v\in V\left(\Omega \right)\;\int_{\Omega }\tau \cdot \varepsilon \left(v\right)dx=f\left(v\right)\right\},$$

where $E_{s}^{2}$ is the set of second rank symmetric tensors, $\varepsilon \left(v\right)\in E_{s}^{2}$ is the symmetric part $\varepsilon_{ij}\left(v\right)=\frac{1}{2}\left(\frac{\partial v_{i}}{\partial x_{j}}+\frac{\partial v_{j}}{\partial x_{i}}\right)$ of the gradient ∇v of the vector field **v** and $\tau \cdot \varepsilon =\tau_{ij}\varepsilon_{ij}$ is the scalar product.

According to Castigliano’s theorem the compliance is expressed by

(8) $$℘\left(k,\mu \right)=\underset{\tau \in \;\Sigma \left(\Omega \right)}{\min}\int_{\Omega }\tau \cdot C^{-1}\tau \;dx,$$

The stress-based version of the IMD method of minimizing the compliance reads (see, e.g., [47,48]): find the fields (k*, μ*) ∈ ℜ, minimizing Compliance (8), or

(9) $$\left(k^{*},\mu^{*}\right)=\underset{\left(k,\mu \right)\in ℜ}{\arg\min}℘\left(k,\mu \right),$$

Let us introduce a norm of a symmetric tensor $\sigma \in E_{s}^{2}$

(10) $$\left|\;\cdot \;\right|:E_{s}^{2}\to R\;,\;\left|\sigma \right|=\frac{1}{\sqrt{2}}\left|\mathrm{tr}\;\sigma \right|+\sqrt{2}\left\|\mathrm{dev}\;\sigma \right\|,$$

where $\mathrm{tr}\;\sigma =\sigma_{ii}$, $\mathrm{dev}\;\sigma =\sigma -\frac{1}{2}\left(\mathrm{tr}\;\sigma \right)I$ and $\left\|\sigma \right\|=\sqrt{\sigma_{ij}\sigma_{ij}}$ are the trace, deviator and Frobenius norm of the tensor $\sigma \in E_{s}^{2}$, respectively. Note that the operation | ⋅ | means the absolute value for a scalar argument, and means Norm (10) for an argument being the 2nd rank tensor, which should not lead to misunderstandings. The most important result of the IMD method is the following:

**Theorem** **1.** *The fields*

(11) $$k^{*}=\frac{\Lambda }{2\sqrt{2}}\frac{\left|\mathrm{tr}\;\tau^{*}\right|}{\int_{\Omega }\left|\tau^{*}\right|dx}\;,\;\mu^{*}=\frac{\Lambda }{2\sqrt{2}}\frac{\left\|\mathrm{dev}\;\tau^{*}\right\|}{\int_{\Omega }\left|\tau^{*}\right|dx},$$

*are solutions of Problem (9), where* $\tau^{*}\in \Sigma \left(\Omega \right)$ *is the solution to the following auxiliary minimization problem:*

(12) $$\tau^{*}=\underset{\tau \;\in \;\Sigma \left(\Omega \right)}{\arg\min}\int_{\Omega }\left|\tau \right|dx.$$

Proof of this theorem for 3D case can be found in [47]. The optimal fields of the Young’s modulus E* and the Poisson ratio ν* can be found from the following formulae:

(13) $$E^{*}=\frac{4k^{*}\mu^{*}}{k^{*}+\mu^{*}}=2H^{*}\;,\;\nu^{*}=\frac{k^{*}-\mu^{*}}{k^{*}+\mu^{*}},$$

where $H^{*}=2/\left(1/k^{*}+1/\mu^{*}\right)$ is the harmonic mean of the moduli k* and μ*.

In the most typical case, there hold: E* > 0 and −1 < ν* < 1, which corresponds to the case of the moduli k* and μ* being positive. On the other hand, in the most degenerated case both the moduli k* and μ* vanish, which determines the subdomains with no material; in this domain both the moduli E* and ν* are undefined. The degenerated materials of extreme values of the Poisson ratio: ν* = −1 and ν* = 1 correspond to the subdomains with k* = 0, μ* > 0 and k* > 0, μ* = 0, respectively. In both the cases the optimal Young’s modulus E* vanishes (see first formula in Equation (13)), but the material does exist. The possibility of the emergence of such degenerated materials results directly from the mathematical formulation of the stress-based version of the IMD method. Introduction of the additional point-wise constraints *k*(x) ≥ *k_min_* and *μ*(x) ≥ *μ_min_*, where *k_min_* > 0, *μ_min_* > 0 are given and fixed, could prevent from the emergence of the mentioned degenerated optimal materials, but then the simplicity of the IMD method would be lost and the Equation (11) cease to hold.

**Remark** **1.** *The 2D version of IMD discussed here applies to both the plane stress problem and to the plane strain problem. In the plane stress problem, a thin plate of thickness h is considered, with the load applied in-plane, with the stress field σ standing for the stress resultants across the plate thickness. If the material of the plate is characterized by the moduli* $E_{m}^{*},\;\nu_{m}^{*}$, then the moduli k*, μ* of the 2D model are expressed by

$$k^{*}=\frac{E_{m}^{*}h}{2\left(1-\nu_{m}^{*}\right)}\;,\;\mu^{*}=\frac{E_{m}^{*}h}{2\left(1+\nu_{m}^{*}\right)}$$

*hence*

$$E_{m}^{*}=E^{*}/h\;,\;\nu_{m}^{*}=\nu^{*}$$

where E*, ν* are given by Equation (13). Just these formulae show how to recover the plane stress problem results from the 2D version of the IMD method. In the plane strain problem the plane domain is viewed as the section of a body of infinite length in the direction orthogonal to the x and y axes. Let k*, μ* are optimal moduli given by Equation (11). Then, the optimal moduli $k_{m}^{*},\;\mu_{m}^{*}$ *of the 3D body are*

$$k_{m}^{*}=k^{*},\;\mu_{m}^{*}=\mu^{*}$$

Thus, the optimal moduli $E_{m}^{*},\;\nu_{m}^{*}$ of the 3D body are given by

$$E_{m}^{*}=\frac{9k^{*}\mu^{*}}{3k^{*}+\mu^{*}}\;,\;\nu_{m}^{*}=\frac{3k^{*}-2\mu^{*}}{2\left(3k^{*}+\mu^{*}\right)}$$

The other alternative is to assume that the optimal material is of cubic symmetry, which is called the Cubic Material Design (CMD), proposed in [53]. Let us briefly summarize the theoretical results of this method (see also [59,60]).

The design variables in two-dimensional version of CMD method are two mutually orthogonal unit vector fields (**m**(*x*), **n**(*x*)) and three elastic moduli (*a*(*x*), *b*(*x*), *c*(*x*)), *x* ∈ Ω involved in Walpole’s [58] representation of a non-homogeneous Hooke tensor **C** of cubic symmetry in the 2D setting:

(14) C(x)=a(x) J+b(x) L(x) +c(x)M(x) , x∈Ω,

where the projectors are

(15) $$\begin{array}{l} J=\Lambda_{1}=\frac{1}{2}I⊗I\;,\;L=\mathrm{II}-S,\;M=S-J,\;S=n⊗n⊗n⊗n+m⊗m⊗m⊗m,\\ I=\delta_{ij}e_{i}⊗e_{j},\;\mathrm{II}=\frac{1}{2}\left(\delta_{ik}\delta_{jl}+\delta_{il}\delta_{kj}\right)e_{i}⊗e_{j}⊗e_{k}⊗e_{l}. \end{array},$$

The design variables (*a*, *b*, *c*) are subject to the same cost (isoperimetric) condition (Equation (4)), where now tr C=a+b+c.

If **m** = **e**_1_, **n** = **e**_2_, then

(16) $$C_{1111}=(a+c)/2,{\;C}_{2211}=C_{1122}=(a-c)/2,C_{2222}=(a+c)/2,C_{1212}=b/2,$$

and other components can be found by the known symmetry conditions, or they vanish.

The stress-based version of the CMD method of minimizing the compliance reads: find the fields (a*, b*, c*) ∈ ℜ, and orthogonal trajectories of the vector fields (m*, n*) at each point *x* ∈ Ω minimizing Compliance (8) or

$${℘}^{*}=\underset{\mathrm{admissible}\;C}{\min}\;\underset{\tau \in \;\Sigma \left(\Omega \right)}{\min}\int_{\Omega }\tau \cdot C^{-1}\tau \;dx,$$

for a cubic Hooke tensor (Equation (14)). The optimal moduli of the cubic material are expressed by:

**Theorem** **2.** *The fields*

(17) $$a^{*}=\frac{\Lambda }{\sqrt{2}}\frac{\left|\mathrm{tr}\;\tau^{*}\right|}{Z^{*}}\;,\;b^{*}=0\;,\;c^{*}=\Lambda \frac{\left\|\mathrm{dev}\;\tau^{*}\right\|}{Z^{*}}$$,

*are solutions of the above problem, where* $\tau^{*}\in \Sigma \left(\Omega \right)$ *is the solution to the following auxiliary minimization problem:*

(18) $$Z^{*}=\underset{\tau \;\in \;\Sigma \left(\Omega \right)}{\min}\int_{\Omega }\left(\frac{\sqrt{2}}{2}\left|\mathrm{tr}\;\tau \right|+\left\|\mathrm{dev}\;\tau \right\|\right)dx$$.

*Moreover, the optimal vector fields* (m*, n*) *follow the trajectories of principal stresses* τ* (see [53]).

A numerical algorithm based on solving the two auxiliary problems (Equations (12) and (18)) has been put forward in [47,49,50]. The trial stress fields τ = τ(*x*) are interpolated by bi-linear polynomials. A detailed description of the numerical methods to be used has been given in the mentioned papers; hence, there is no need to repeat the algorithm here.

The common property of the least compliant designs formed by the IMD and CMD methods is a local boundedness of a certain norm of strain (cf. [51,53]). Consequently, the magnitudes of the optimal moduli follow the values of stresses. Let us stress that this process is not iterative, since the auxiliary problems (Equations (12) and (18)) are material independent.

For the future purposes, to have a better insight into the IMD and CMD solutions let us compare the 2D matrix representations of Hooke’s tensors for the isotropic material and for the cubic material observed in the frame coinciding with the axes of the principal material directions. Let us start with recalling the 2D matrix representation of a 4th rank symmetric tensor **C** = (*C_ijkl_*) (cf. [47], Equation II.4).

(19) $$E=\left[\begin{array}{ccc} C_{1111} & C_{1122} & \sqrt{2}C_{1112}\\ C_{1122} & C_{2222} & \sqrt{2}C_{2212}\\ \sqrt{2}C_{1112} & \sqrt{2}C_{2212} & 2C_{1212} \end{array}\right].$$

The constitutive matrix **E**^I^ of the isotropic tensor **C**^I^ is expressed in terms of the bulk and shear moduli (*k*, *μ*), or in terms of the Young’s modulus and the Poisson ratio (*E*, *ν*) as follows

(20) $$\begin{array}{cc} E^{I}=\left\{\begin{array}{ccc} k+\mu & k-\mu & 0\\ k-\mu & k+\mu & 0\\ 0 & 0 & 2\mu \end{array}\right\} & =\;\frac{E}{1-\nu^{2}}\;\left\{\begin{array}{ccc} 1 & \nu & 0\\ \nu & 1 & 0\\ 0 & 0 & \left(1-\nu \right) \end{array}\right\} \end{array}.$$

The constitutive matrix **E**^C^ representing the cubic symmetry tensor **C**^C^ referred to its principal directions is expressed in terms of the moduli involved in Equation (14)

(21) $$\begin{array}{c} E^{C}=\left\{\begin{array}{ccc} a/2+c/2 & a/2-c/2 & 0\\ a/2-c/2 & a/2+c/2 & 0\\ 0 & 0 & b \end{array}\right\} \end{array}.$$

By analogy with isotropy, we introduce now the notation: *k* = *a*/2, *μ* = *c*/2, *b* = 2α*μ*, which should not lead to misunderstandings. The representation (Matrix (21)) assumes the form

(22) $$\begin{array}{c} E^{C}=\left\{\begin{array}{ccc} k+\mu & k-\mu & 0\\ k-\mu & k+\mu & 0\\ 0 & 0 & 2\alpha \mu \end{array}\right\} \end{array},$$

very similar to the isotropic one; for *α* = 1 both Matrices (20) and (22) coincide. We use the notation: *k*, *μ*, but these are now not bulk and shear moduli; the latter notions are used only for isotropy. Nevertheless, for this frame of observation we can introduce the Young’s modulus and Poisson ratio by Equation (13), still denoted by *E* and *ν.*

Rotation of the tensor **C**^C^ by 45° gives the matrix representation as below:

(23) $${\tilde{E}}^{C}=\left\{\begin{array}{ccc} k+\tilde{\mu } & k-\tilde{\mu } & 0\\ k-\tilde{\mu } & k+\tilde{\mu } & 0\\ 0 & 0 & 2\beta \tilde{\mu } \end{array}\right\},$$

where the modulus *k* is kept unchanged $\tilde{\mu }=\alpha \mu$ and, *β* = 1/*α*.

The standard form of the matrix **E**^C^ will be assumed as in Equation (22), where 0 ≤ *α* ≤ 1.

## 3. Optimum Design of a Deep Beam by the IMD Method

**Figure 1:**
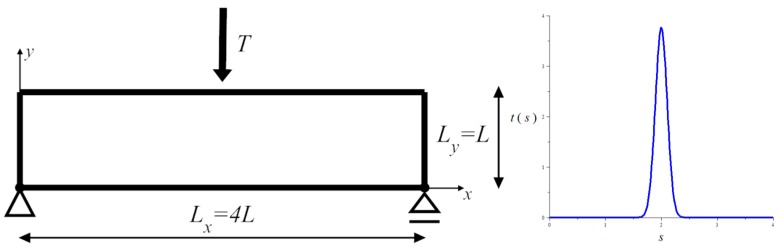
The plate supported at two bottom corner nodes and loaded with one vertical force applied to the upper face (**left**); and modeled by a smoothing weight function (**right**).

The example concerns the plate-like 2D body of length *L_x_* = 4*L* and height *L_y_* = *L*, *L* being a referential length (equal to 1 m in calculations), simply supported at bottom corners and subjected to the in-plane vertical force *T* = −*T* **e**_2_ centered on the upper edge (see Figure 1). The right support has the possibility of free horizontal displacement and the loading *T* is represented by the traction **t** = (0, *t*) modeled by a smoothing weight function, i.e., $T=\int_{0}^{L_{x}}t\left(s\right)\;ds≅1.0\left[N\right]$, $t\left(s\right)=t_{\max}\exp\left(-{\left(\left(s-L_{x}/2\right)/w\right)}^{2}\right)$, $t_{\max}≅3.76\;\left[N/m\right]$, w=0.15 (see Figure 1). The finite element mesh used is composed of *E_x_* × *E_y_* = 100 × 25 = 2500 bilinear finite elements C2D4. The value of the referential Young’s modulus *E*_0_ is assumed equal to 1.0 N/m^2^.

**Figure 2:**
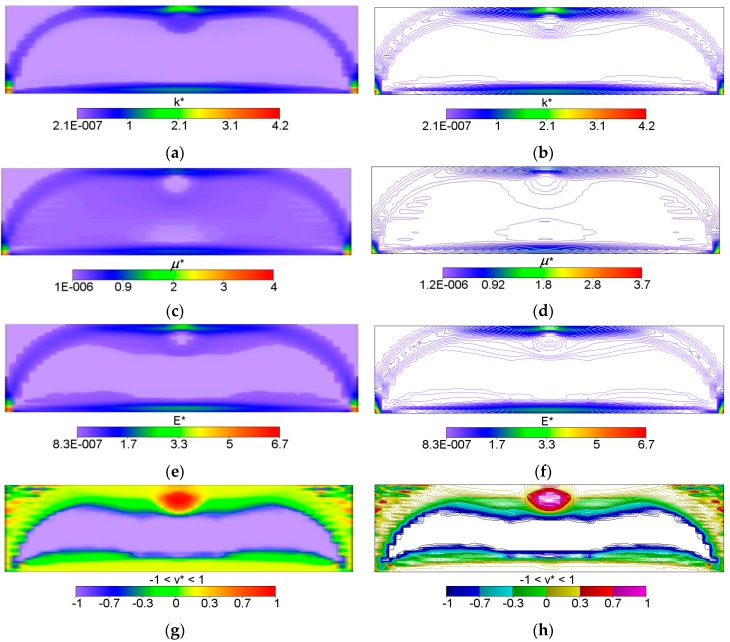
First six figures: Volume-render (**left**); and contour (**right**) views of the optimal and relative (i.e., divided by *E*_0_) layouts of (**a**,**b**) bulk k*; (**c**,**d**) shear μ* and (**e**,**f**) Young’s E* moduli, respectively. The last two figures: (**g**) Volume-render and (**h**) contours views of optimal distribution of the optimal Poisson’s ratio ν*.

The IMD confirms here its main feature of the method capable of solving simultaneously the shape design problem and the material distribution problem. The shape of the optimal body is determined as the effective domain of the minimizer of Problem (12). The optimal body is thus cut out from the given rectangular design domain; both the upper corners turn out to be unnecessary, hence the optimal body assumes the shape of the domain where both or one of the moduli: the bulk modulus k* and shear modulus μ* assume positive values (see Figure 2). The largest values of the moduli concentrate along the strips: along the lower face, along the arch-like strips and the upper strip shorter than the lower one (cf. Figure 2).

**Figure 3:**
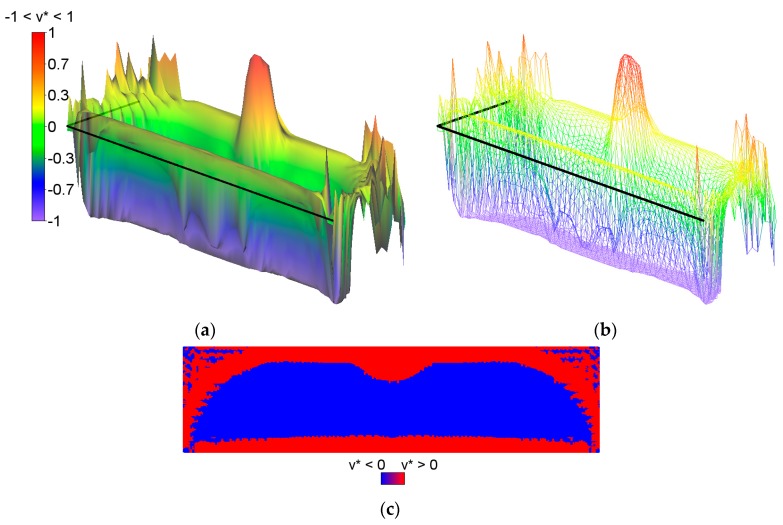
Two first figures: (**a**) The height field view and (**b**) the scatter plot view of the distribution of the optimal Poisson ratio ν*; the negative values are shown in blue color and the positive values are shown in red. The third figure (**c**) shows the domains where the optimal Poisson ratio ν* assumes positive and negative values.

**Figure 4:**
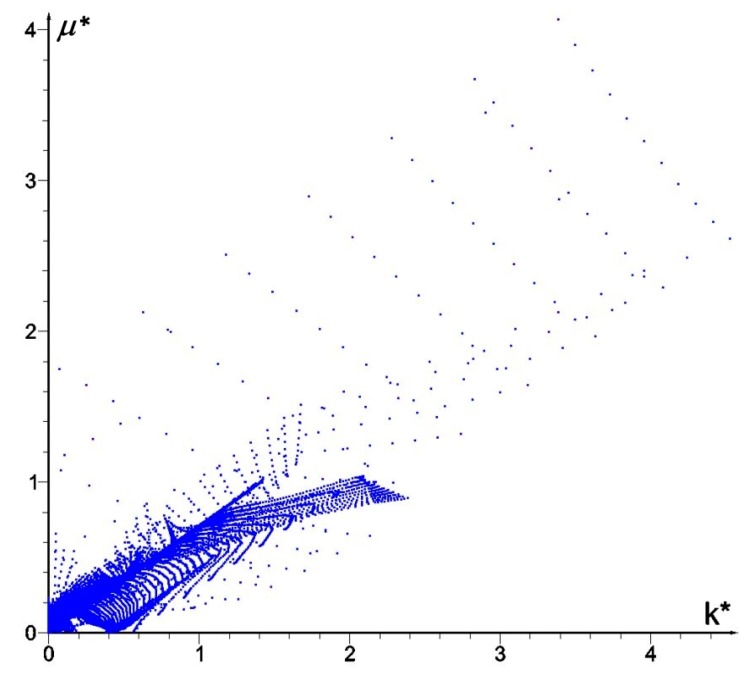
Distribution of optimal pairs of points (k*, μ*).

The bulk modulus k* and shear modulus μ* reach the values within the range from about ~2.0·10^−7^ to ~4.0. Inside the inner subdomain bounded by the strips mentioned above, the optimal modulus k* reaches close to zero values, but this observation does not apply to the optimal modulus μ*, which reaches also small but positive values, hence there a degenerated material emerges with the extreme negative value of Poisson’s ratio ν* equal to −1. It is also worth noting that below the applied load, a characteristic “bubble” subarea emerges in which the optimal Poisson’s ratio ν* reaches a maximum positive value equal to 1 (red and violet color in Figure 2g,h, respectively). This is due to the fact that the optimal bulk modulus k* is positive, and the optimal shear modulus μ* is equal (or close) to zero, so according to the second formula in Equation (13), the degenerated material once more emerges with extreme positive value of Poisson’s ratio ν* equal to 1. At the same time, in the subdomains on the left and right side of the “bubble”, an auxetic material with a Poisson’s ratio value of about −0.3 emerges (green color). Under these subdomains the Poisson ratio decreases rapidly to negative values: from −0.7 (blue color) to −1 (violet color) (see Figure 3). This phenomenon is known; it is called the indentation behavior of auxetic materials, which improves indentation resistance when compared to conventional materials (see [5,6]). In the example considered, the reason of this phenomenon is that the local increasing of the indentation resistance contributes to the decreasing of the compliance, i.e., decreasing of the work done by the loading **T** on the displacement **u**. Note that the region ν* = 0 degenerates to a contour dividing the subdomains corresponding to ν* < 0 and ν* > 0 (see Figure 3c). The distribution of the optimal moduli k*, μ* shows the appearance of the auxetic material (k* < μ*), cf. Figure 4.

## 4. Optimum Design of a Deep Beam by the CMD Method

**Figure 5:**
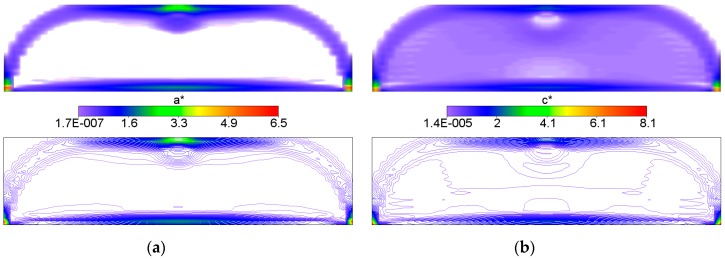
(**a**) Volume-render and (**b**) contours views of the optimal and relative (i.e., divided by *E*_0_) layouts of a* and c* moduli, respectively.

**Figure 6:**
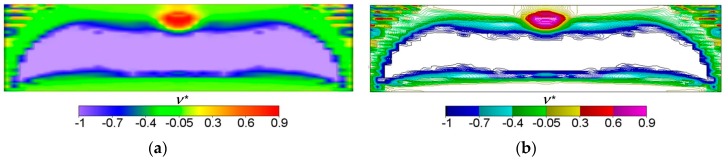
(**a**) Volume-render and (**b**) contour views of optimal distribution of the optimal Poisson’s ratio ν*.

**Figure 7:**
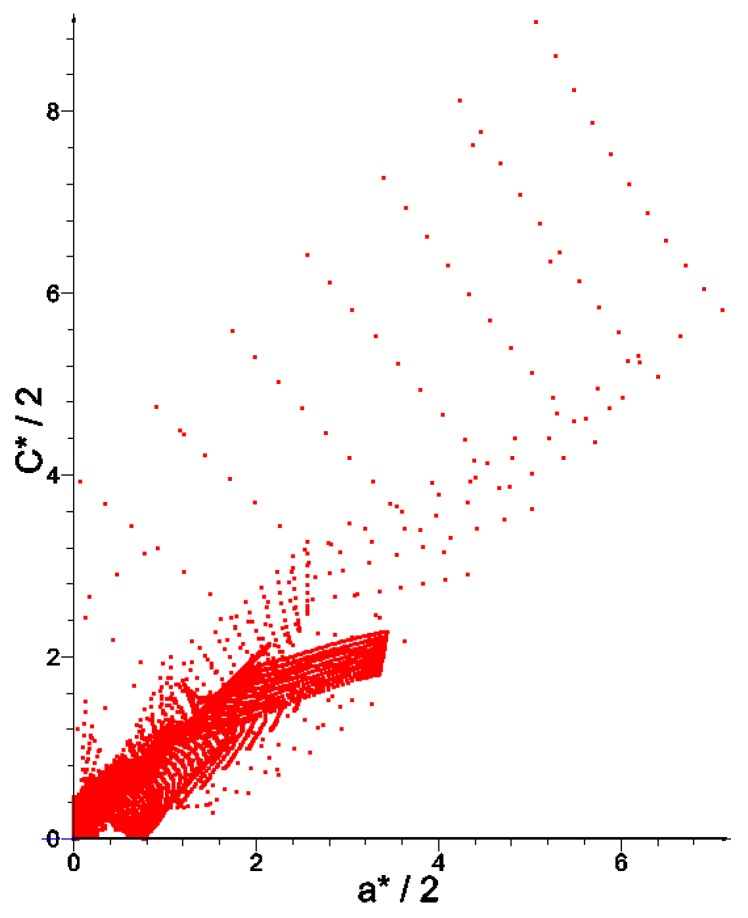
Distribution of optimal pairs of points (a*/2, c*/2).

**Figure 8:**
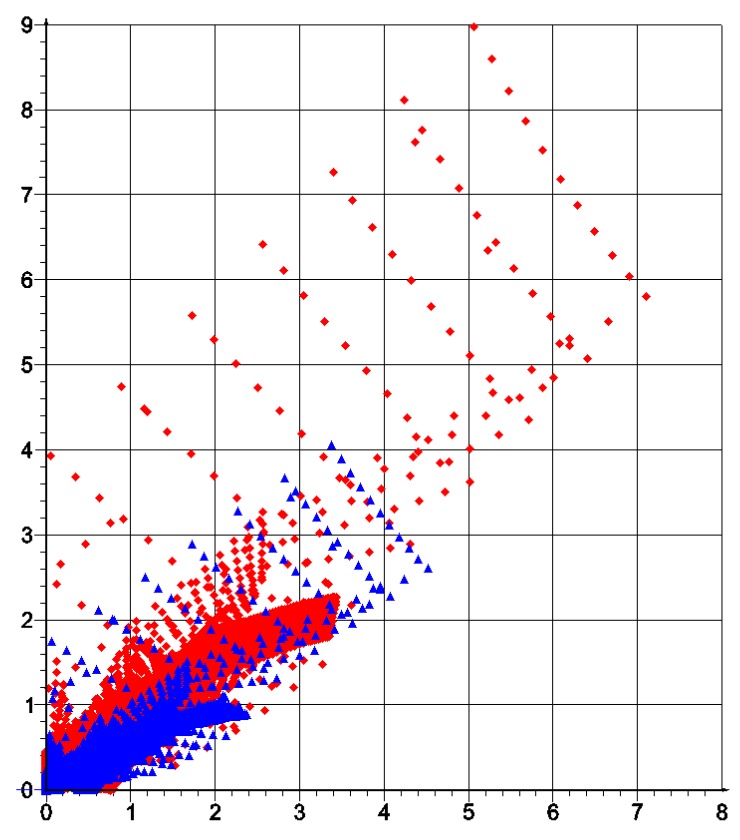
Distribution of optimal pairs of points (k*, μ*) (blue triangles) and (a*/2, c*/2) (red diamonds) for IMD and CMD method, respectively.

The same beam is designed now from a cubic material by using the CMD method outlined in Section 2. An arbitrary cubic material is characterized by three independent elastic moduli: *a*, *b*, *c*, but the process of the compliance minimization results in vanishing the modulus *b* (see Equation (17)). As indicated in Section 2 for the cubic material phase one can assign Poisson’s ratio as equal to ν = (*a* − *c*)/(*a* + *c*). As proven in [53], the optimum design leads to the stress and strain trajectories coinciding with the main material axes. If referred to this frame, we obtain the identity *C*_1212_ = 0, which means that the underlying microstructures should have zero stiffness due to shear. The optimal cubic material forming the stiffest deep beam of Figure 1 satisfies the mentioned properties. The layout of the optimal moduli a*, c* is similar (see Figure 5), but not identical with the layout of *k*, *μ* of the isotropic design of Figure 2. However, in the cubic design, one can note the subdomains of negative Poisson ratio (see Figure 6). Distributions of optimal pairs of points (a*/2, c*/2) are shown in Figure 7. Since the moduli (a*/2, c*/2) are counterparts of the moduli (k*, μ*) it is worth showing the results of the IMD and CMD methods in one figure (see Figure 8). We note that CMD results lie in a broader domain. To explain it let us note that the optimum design method IMD involves two unknown fields, the method CMD involves three unknown fields, while the same compliance functional is minimized. Consequently, the IMD method imposes stronger constraints thus delivering the results lying in a narrower domain than the CMD results.

## 5. Effective Properties of Periodic Composites—Numerical Homogenization

Prior to considering the problem of recovery of two-phase microstructures of effective properties predicted by the IMD method it is helpful to recall the homogenization formulae for 2D elasticity (cf. Sanchez-Palencia [61] or [62]). Although the theorems of the homogenization theory refer to arbitrary inhomogeneity, its useful formulae concern the composites of the repetitive properties for which the RVEs are identified with the periodicity cells Y*_e_*, *e* being a small parameter. Upon rescaling we work with the basic cell Y of a rectangular shape parameterized by the (*y*_1_, *y*_2_) Cartesian system. The elastic moduli of the basic cell are still denoted by *C_ijkl_*, but they are viewed as functions of argument *y = (y*_1_, *y*_2_); with *y* being a point of Y. The basic cell problem of the homogenization theory reads:

Find the Y-periodic vector fields $\chi^{\left(11\right)}\left(y\right),\chi^{\left(12\right)}\left(y\right)=\chi^{\left(21\right)}\left(y\right),\chi^{\left(22\right)}\left(y\right)$ such that

(24) $$\int_{Y}C_{ijpq}\left(\delta_{pk}\delta_{ql}+\frac{\partial \chi_{p}^{kl}\left(y\right)}{\partial y_{q}}\right)\;\frac{\partial v_{i}(y)}{\partial y_{j}}dY=0\;,$$

holds for any Y-periodic vector fields **v** defined on Y.

The gradients of $\chi^{\left(11\right)}\left(y\right),\chi^{\left(12\right)}\left(y\right)=\chi^{\left(21\right)}\left(y\right),\chi^{\left(22\right)}\left(y\right)$ are uniquely determined, even if the moduli *C_ijkl_* suffer jumps within Y. Having solved Problem (20) one can define the homogenized moduli by

(25) $$C_{ijkl}^{H}=\frac{1}{\left|Y\right|\;}\int_{Y}C_{ijpq}\left(\delta_{pk}\delta_{ql}+\frac{\partial \chi_{p}^{kl}\left(y\right)}{\partial y_{q}}\right)\;dY.$$

The tensor **C**^H^ thus constructed is invariant with respect to translations and rotations of the periodicity cell Y.

The numerical homogenization (for details, see, e.g., [63]) reduces to solving Equation (24) by the finite element method (FEM) or by other appropriate numerical approach. Using FEM requires a proper construction of the mesh Y*_k_* of Y to make it possible to satisfy the periodicity conditions involved in Equation (24). The simplest FE algorithms start from the approximation of the test fields **v** = **Nq**, leading to the strain approximation of the form ε(v)=Bq, **N** being the matrix of the shape functions, and **B** being defined according to the definition of strain. The periodicity conditions are fulfilled by identifying the nodes at opposite edges, by virtue of the mesh being properly introduced. For the *k*-th element we define the matrices

(26) $$K_{k}=\int_{Y_{k}}B_{k}^{T}E_{k}B_{k}\;dY,\;H_{k}=\int_{Y_{k}}B_{k}^{T}E_{k}\;dY,$$

and then aggregate them to build: *K* = Σ *K_k_* being the FE stiffness matrix of *Y* and *H* = Σ *H_k_* being the matrix comprising the self-equilibrated pseudo load vectors. The components of these pseudo load vectors are determined by the layout of the elastic moduli within *Y*; they vanish if the layout of the elastic properties is homogeneous within *Y*.

The matrix representation of the homogenized moduli tensor is given by

(27) $${Ε}^{H}=\;\frac{1}{\left|Y\right|}\left(\int_{Y}EdY-H^{T}K^{-1}H\right).$$

## 6. Recovery of the Underlying Microstructures by the Combinatorial Homogenization-Based Process

The IMD method delivers the layouts of the moduli k*(*x*), μ*(*x*) within Ω. Given two isotropic phases, called further M0 and M1 of moduli *k*_0_(*x*), *μ*_0_(*x*) and *k*_1_(*x*), *μ*_1_(*x*) we state the problem of recovery the two-phase microstructure within Y(*x*) such that the homogenized tensor **C**^H^ is isotropic of moduli *k*^H^(*x*), *μ*^H^(*x*), coinciding with the moduli k*(*x*), μ*(*x*). Let us note that the moduli *k*_0_(*x*), *μ*_0_(*x*) and *k*_1_(*x*), *μ*_1_(*x*) must be appropriately chosen once and then should serve for each x. One of the choices is *k*_0_(*x*) = 0, *μ*_0_(*x*) = 0, which means that the problem reduces to finding the layout of the isotropic material of moduli *k*_1_(*x*), *μ*_1_(*x*) within Y(*x*) to achieve given moduli k*(*x*), μ*(*x*) by the homogenization method.

The CMD method delivers the layouts of the moduli k*(*x*), μ*(*x*), α*(*x*). We shall recover the two-phase cubic microstructures made of the isotropic phases of the moduli *k*_0_(*x*), *μ*_0_(*x*) and *k*_1_(*x*), *μ*_1_(*x*) given a priori. The unknown is the layout of these phases within the cell Y(*x*) such that the resulting homogenized tensor **C**^H^ is of cubic symmetry and is characterized by the moduli *k*^H^(*x*), *μ*^H^(*x*), *α*^H^(*x*) coinciding with k*(*x*), μ*(*x*), α*(*x*). The final computational results will refer to the case of the material M0 being a void.

**Figure 9:**
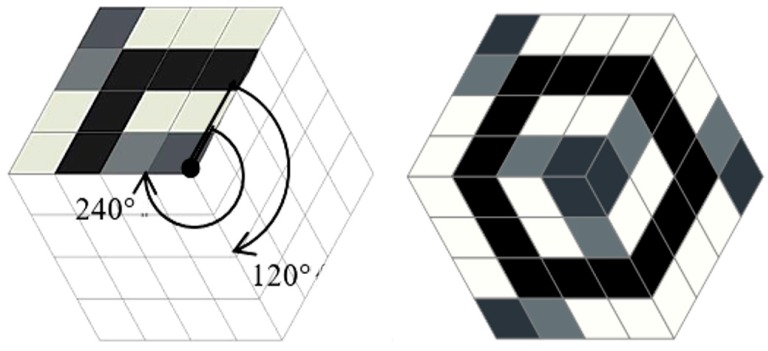
Basic structure and the cell of periodicity Y^I^ exhibiting homogenized exact isotropic properties of a periodic body.

To achieve isotropy of the homogenized tensor we adopt the Y^I^ cells as hexagons constructed by two subsequent rotations of a basic material domain by the angle 120° (see Figure 9). The periodicity assumptions are imposed by identifying the degrees of freedom at the opposite sides of the hexagon. Irrespective of the properties of the rotated part, the resulting homogenized tensor is characterized by all conditions of isotropy. This method has been proposed in [64].

**Figure 10:**
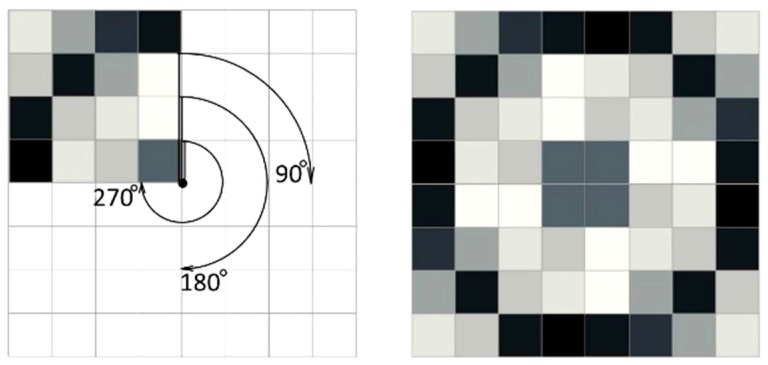
Basic structure and cells of periodicity Y^C^ exhibiting homogenized exact cubic symmetry properties of a periodic body.

To achieve cubic symmetry, the procedure is even simpler: it is sufficient to rotate three times a square material domain by the angle 90° (see Figure 10). This rotated domain is called the basic material domain, as before. The periodicity conditions are fulfilled by identifying the degrees of freedom at the opposite edges of the final square cell Y^C^.

**Figure 11:**
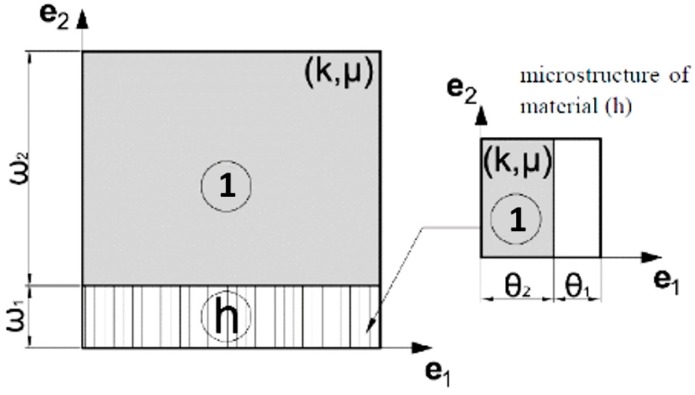
Microstructure with cubic symmetry properties of **C** proposed in [65]. Material 2 is isotropic, white is void. Courtesy of the authors. (Reproduced with permission from Engineering Transaction; published by Institute of Fundamental Technology Research Polish Academy of Sciences, Warsaw, National Engineering School of Metz, and Poznan University of Technology, 2017.)

In the case of the cubic design, trying to fulfill the optimum design result b* = 0 (see Equation (17)), we face a difficult problem of constructing the microstructure of this kind of the degeneracy. This modulus reflects a stiffness due to shear. To attain this special property one can apply the two-stage layering construction with using an isotropic material and voids, as proposed in [65]. This lamination construction is shown in Figure 11. However, in the present paper, we confine the designing to first rank microstructures; consequently, this mathematical construction will not be used. 

In the present paper we shall show at selected points more practical 1st rank microstructures for which the moduli *a*^H^, *c*^H^ are almost equal to a*, c* (found by the CMD method), while the additional condition of b* being zero will be omitted. Nevertheless some microstructures appear to have very small b*, which will be discussed in Section 7.2.

## 7. Analysis of the Ranges of the Effective Moduli of the Isotropic and Cubic-Symmetry Composites. Selecting Microstructures of Extremal Properties

The porous composites will be discussed, the void phase being viewed as the material M0 of the negligible isotropic moduli (*k*_0_, *μ*_0_) = (*k*_1_, *μ*_1_·10^−9^). The isotropic material phase M1 is characterized by (*k*_1_, *μ*_1_) = (0.6667, 0.4). Then the Young’s modulus and the Poisson ratio are (*E*, *ν*) = (1.0, 0.25); the moduli are scaled by the reference modulus *E*_0_ = 11.25.

The homogenization-based process will be performed by a total combinatorial search of the layouts of a single isotropic material within the *basic material domain*. The search is done among all possible positions of the material elements within the *basic material domain* being divided into the elements. The mesh is quite coarse to make the global search possible. The cells Y^I^ and Y^C^ are divided into sixteen eight-node elements (see Figure 9 and Figure 10). To each element, either the material phase M1 (black) or the void phase M0 (white) is assigned. For each possible non-degenerated layouts of the phases, the effective moduli (*k*^H^, *μ*^H^) or (*k*^H^, *μ*^H^, *α*^H^) are computed using Equation (20). The volume fraction of the material is defined as *ρ* = (area of material elements or of the phase M1)/|Y|.

### 7.1. The Isotropic Composites of Negative Poisson’s Ratio

**Figure 12:**
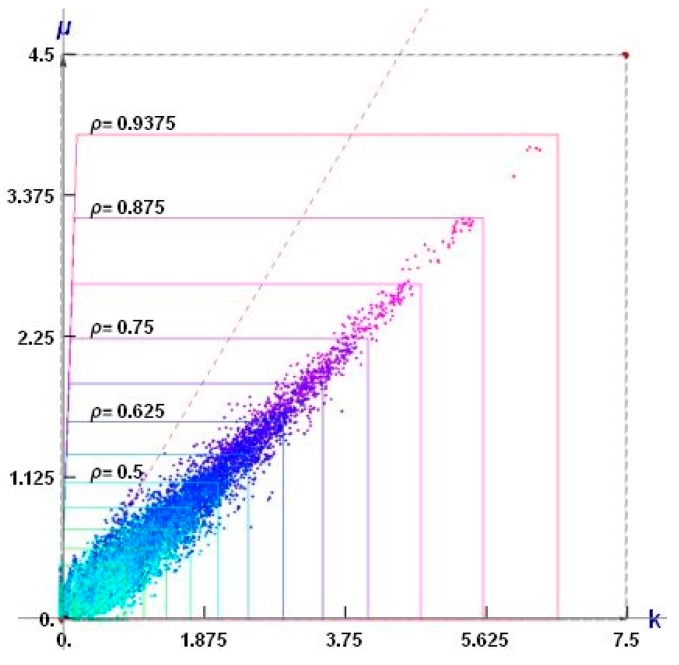
The sets of points of coordinates (*k*^H^, *μ*^H^) corresponding to the isotropic periodic composites of the two-phase cells (see Figure 9) for all possible black and white non-degenerated layouts for the given mesh. The axes are scaled differently, thus changing the Cherkaev–Gibiansky bounds into squares.

The points of coordinates (*k*^H^, *μ*^H^) constitute a cloud of points (see Figure 12). The dashed red line *k* = *μ* separates the composites of the positive and negative Poisson ratio. All results lie within the Cherkaev–Gibiansky bounds (see [66]) being tighter than the Hashin–Shtrikman bounds.

**Figure 13:**
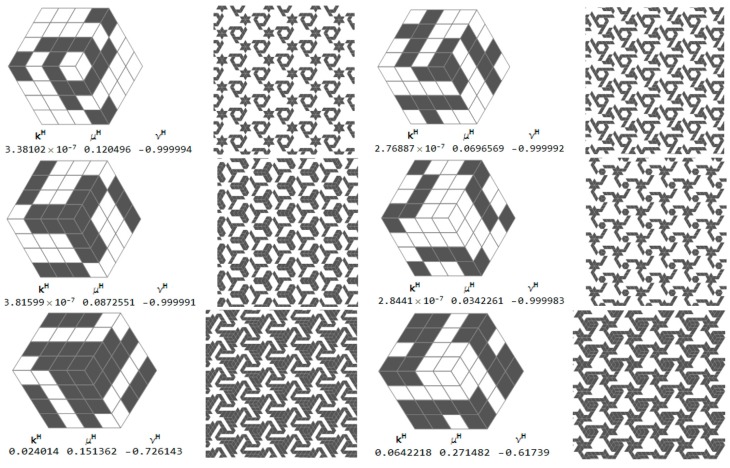

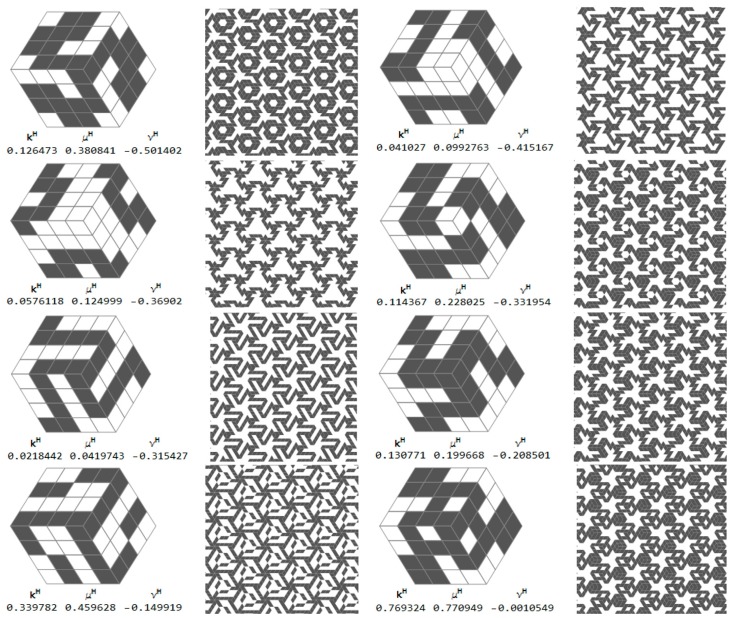
Selected auxetic 1st rank isotropic microstructures and the effective moduli. Cells of periodicity, and the tessellated patterns.

Among thousands of microstructures, one can indicate those of a negative effective Poisson’s ratio *ν*^H^. The microstructures of *ν*^H^ close to −1 are characterized by the modulus *k*^H^ close to zero (cf. Figure 13).

### 7.2. The Cubic Composites of Extremal Properties

**Figure 14:**
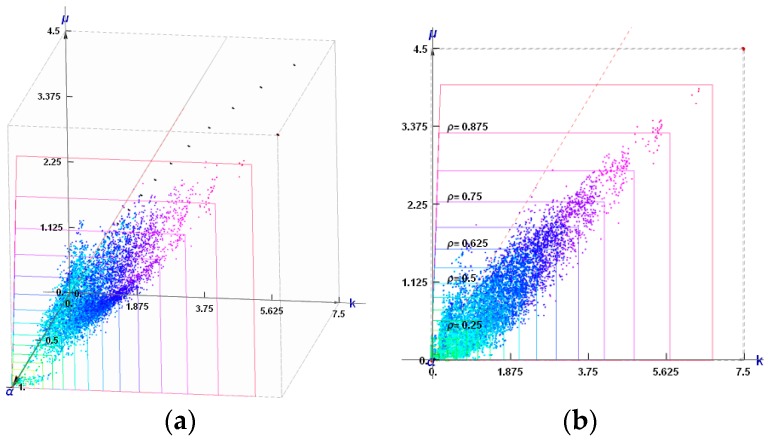
(**a**) The sets of points of coordinates (*k*^H^, *μ*^H^, *α*^H^) corresponding to the periodic composites of cubic symmetry (see the two-phase cell shown in Figure 10). The plane *k* = *μ* is marked by a dashed red line (note that the plane *k* = *μ* is visible as a line) Black points on the plane *α* = 0 refer to microstructures from Figure 11; (**b**) The set of points (*k*^H^, *μ*^H^, *α*^H^) projected onto *α*^H^ = 1 plane.

The points (*k*^H^, *μ*^H^, *α*^H^) constitute a dense cloud (see Figure 14a). Upon projecting them on the plane *α*^H^ = 1, we obtain a plane cloud of results (see Figure 14b). The plane *k = μ* separates the composites of the positive and negative Poisson ratio. All results lie within the Cherkaev–Gibiansky bounds [66], referring to the isotropic composites.

**Figure 15:**
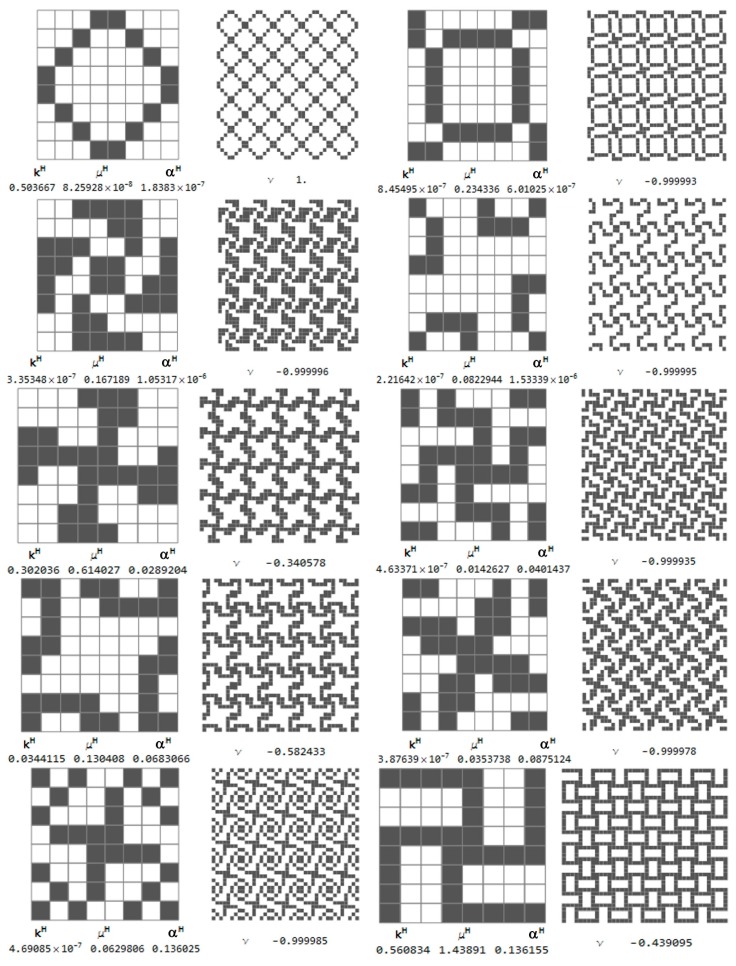

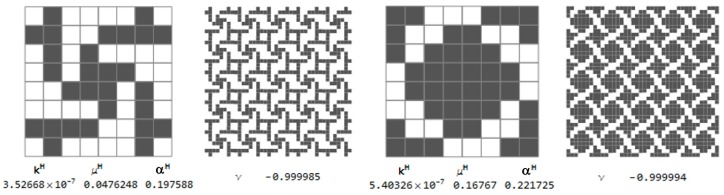
Selected auxetic 1st rank cubic microstructures and the effective moduli. Cells of periodicity, and images upon their tessellated patterns.

The selected microstructures and their effective moduli are shown in Figure 15. One can indicate the microstructures for which ν^H^ is close to 1 and other microstructures for which ν^H^ is close to −1. Some of them are characterized by a very small modulus *b*^H^, but this is not a rule (cf. Figure 15).

## 8. Recovery of the Optimal Microstructure Corresponding to the IMD and CMD Designs

Our aim is to apply the local results shown in Figure 12 and the optimal microstructures shown in Figure 13 for the global optimal design found by IMD (see Figure 4) and to use the local results given in Figure 14 and the cubic microstructures shown in Figure 15 for the global CMD design (see Figure 7). The main observation is that the whole range of the moduli in the optimal designs can only be attained if the material density is small—say smaller than 0.1. Only then one can model all possible ratios of the *k*/*μ* moduli. On the contrary, for the density close to 1 only very specific *k*/*μ* ratios can be achieved. However, basing on low density composites, we must reckon with the buckling of the ligaments and with great magnitudes of stresses, resulting in plastic yield. This question has already a rich literature, but the methods proposed till now have never been used to construct the optimal structures by the IMD or CMD methods.

To choose at each node the optimal microstructure of the smallest error defined by (28) an additional computation of the error for each available patterns is required (see Figure 12 for IMD and Figure 14 for CMD), and the pattern of the smallest error should be chosen.

(28) $$Error={\left(\frac{k^{H}-k^{*}}{k^{*}}\right)}^{2}+{\left(\frac{\mu^{H}-\mu^{*}}{\mu^{*}}\right)}^{2}.$$

**Figure 16:**
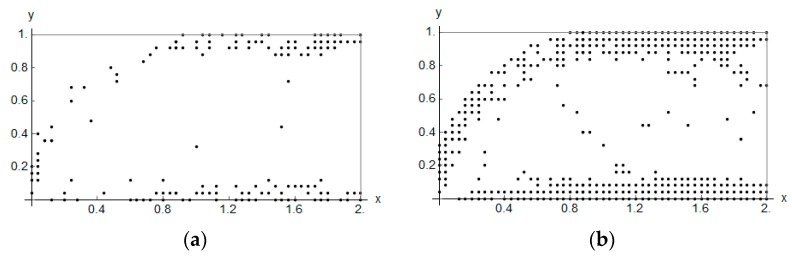

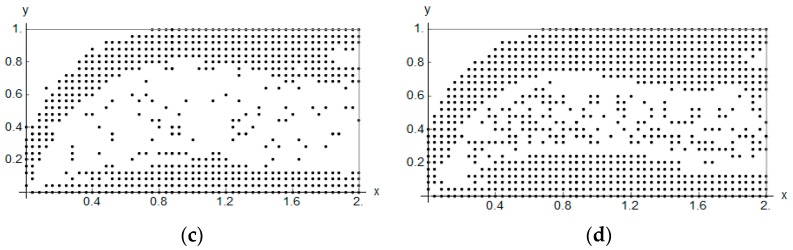
Positions of the isotropic RVEs approximating the IMD results (Figure 2 and Figure 3) with an error (Equation (28)) less than: (**a**) 0.0001; (**b**) 0.001; (**c**) 0.01; and (**d**) 0.1.

**Figure 17:**
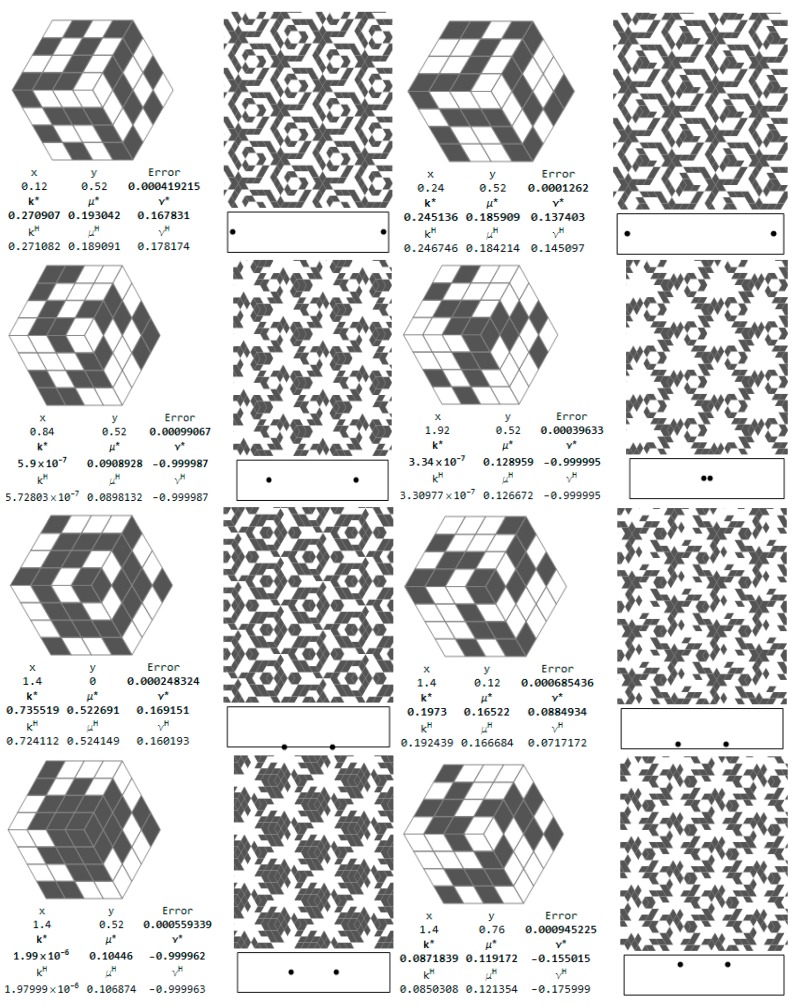

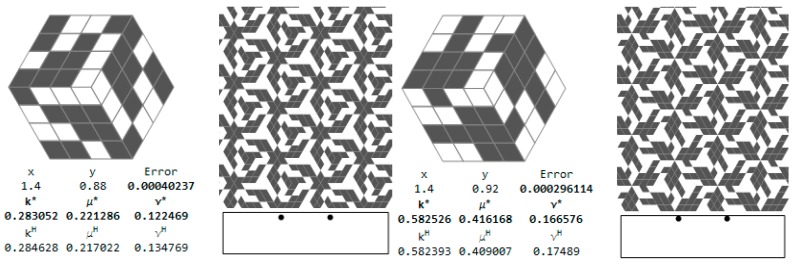
Optimal isotropic microstructures approximating the IMD results with the error (Equation (28)) less than 0.001.

Let us show first the isotropic RVEs for which the approximation error Equation (28) is less than 0.001 (see Figure 16b). The central points of the RVEs lie along the sections: *y* = 0.52 and *x* = 1.4. The coordinates of these points are shown in the figure. Moreover, there are set up: the values of the optimal moduli (k*, μ*) found by the IMD method, the values of the moduli (*k*^H^, *μ*^H^) characterizing the given layout of the phases and the tessellated patterns. The positions of the pairs of the central nodes of the RVEs are shown within the design domain, see Figure 17.

**Figure 18:**
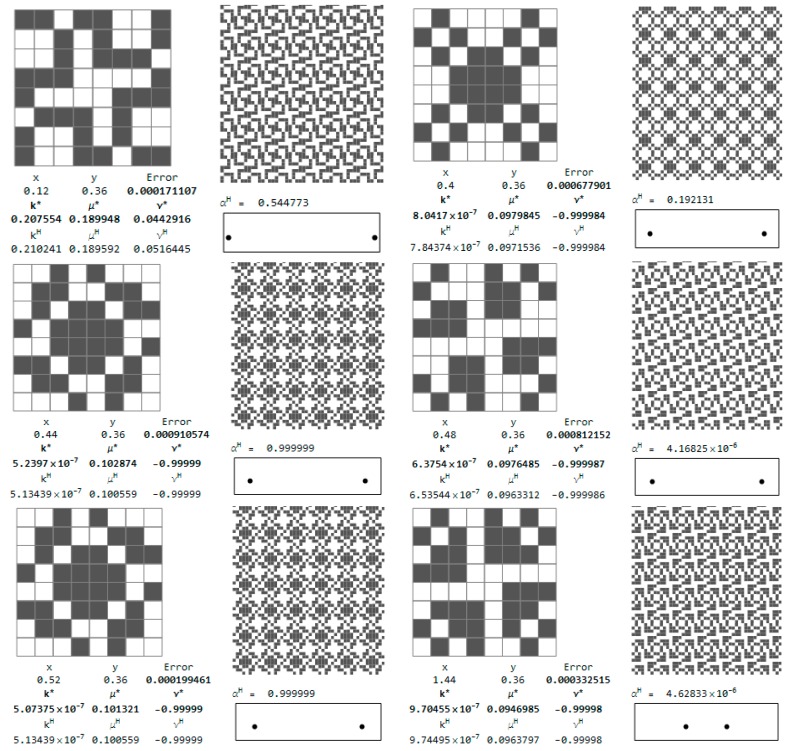

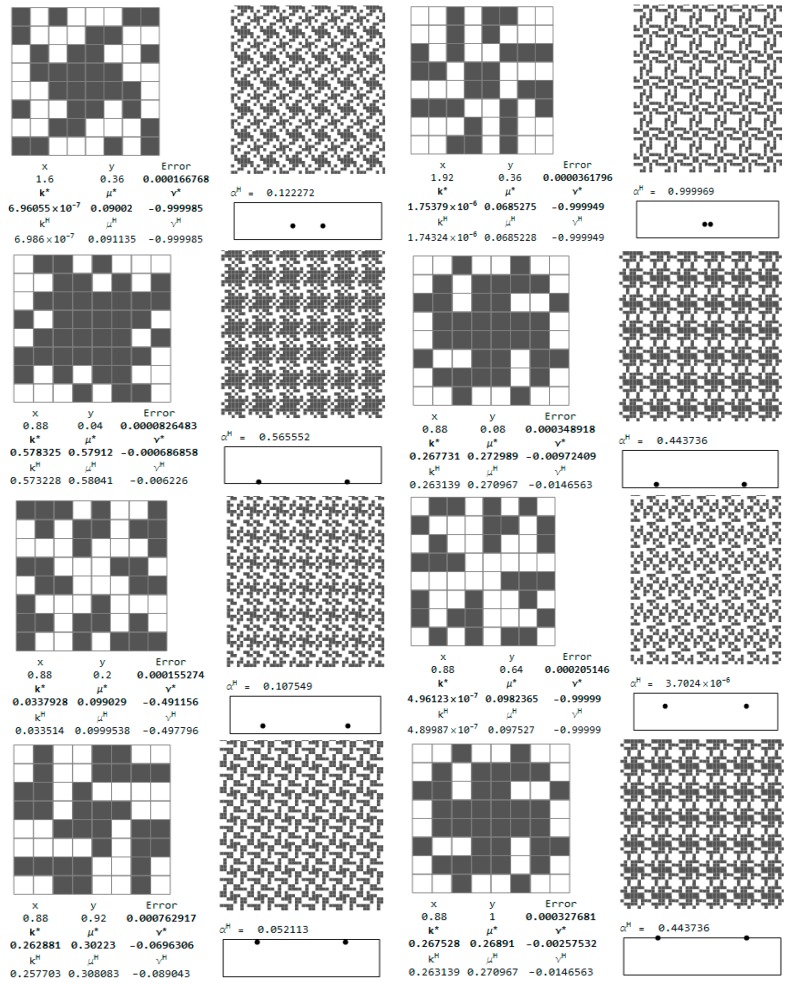
Optimal cubic microstructures approximating the CMD results with the error (Equation (27)) less than 0.001.

Selected cubic RVEs (lying on the sections *y* = 0.36 and *x* = 0.88 for which the error (Equation (28)) is less than 0.001) constructing the CMD optimum design of Figure 5 and Figure 6 are shown in Figure 18. The positions (*x*, *y*) of the RVEs in the design domain are denoted by the pairs of dots. For each microstructure the triplet (*k^H^*, *μ^H^*, *α^H^*) is set up, disclosing that the modulus *α*^H^ usually deviates from α* = 0. The results show that at many points the optimal Poisson’s ratio assumes negative values and can reach the lower bound −1.

## 9. Final Remarks

Deformation of the optimal least compliant elastic bodies made of isotropic or cubic material of spatially varying properties turns out to be point-wise bounded if the unit cost of the designs is assumed as proportional to the trace of the Hooke tensor (see [50,67]). Consequently, the magnitudes of the optimal moduli follow the values of the stress characteristics. In the case of self-equilibrated loads and convex domains, the designer has a control over the stress magnitudes inside the body, hence at least for such cases the material properties may be rationally designed by the IMD and CMD methods used in this work. Let us note that the design space of the CMD method is much greater than that of the IMD method, since isotropy introduces tighter constraints on the final design.

The paper shows the optimal isotropic and cubic 2D microstructures of extreme properties, necessary to fulfill the local stress conditions of the body of the optimal inhomogeneity. The analysis is performed in the plane, yet the 2D isotropic designs (found by the IMD) proposed in the paper may be naturally extended to transverse isotropy in 3D. On the other hand, the 2D cubic designs may be easy extended to 3D cubic designs. Note that the isotropy in 3D is very difficult to mimic by the RVE design; the known sixth rank lamination construction is rather non-manufacturable (see [68]). Thus, the 2D optimal RVEs shown in the paper may be used to design the 3D microstructures showing transverse symmetry or cubic properties. Indeed, the microstructures of the contemporary metamaterials are usually cubic (see [56]).

In the 2D setting discussed, in the case of the absolute value of the trace of the stress tensor being smaller than the Euclidean norm of its deviator, the optimal isotropic material assumes auxetic properties. They will be conveyed to the transverse isotropic bodies designed upon the 2D results.

This paper raises the question of the substantial porosity problem. In fact, a substantial porosity is a very characteristic property of the auxetic materials. This feature refers to a variety of microstructural models at micro, meso and macro levels, regardless of all other well-known properties of these materials. It is therefore fully justified to conclude that “…this type of material is less stiff than the solids from which they are made. …Consequently, for applications that require substantial load-bearing, they are not the best choice.” (See Section 5.1 Limitations on page 146 in [7].) In the present authors’ opinion, this statement can only be accepted in the case of strength and/or buckling analysis limited to the subdomains in which the load is applied locally. The results obtained by the IMD method confirm that the minimization of the work done by applied loading, i.e., the minimization of the compliance, enforces the appearance of smaller or larger subdomains in which the optimal distribution of the bulk and shear moduli results in an auxetic material. Undoubtedly, however, a local loss of stability or failure of the plasticity condition of a microstructure in a very porous medium may become a serious technical problem. This problem was raised in many works (see, e.g., [41]), where the flexibility at the vertices and stress concentration effect are analyzed in detail. The IMD method does not take into account the underlying microstructure, while just designing a realizing microstructure must be based on the stress analysis (see, e.g., [69]). The problem of how to introduce the stress constraints into the optimum design problems of composites and how to determine the lower bound for the trace of the stress tensor at the RVE level was cleared up in [70,71]. The constraints for preventing the local stress reaching the maximal limiting values may be independently introduced point-wise as the Huber–Mises condition (numerically, at least at all Gauss points). Such simulations have already been carried out; the preliminary results are promising and will be published in the forthcoming papers.

The combinatorial method of choosing the optimal microstructures makes it possible to obtain the composites characterized by a relatively small approximation error (Equation (28) only at small number of RVEs. Construction of the RVEs in the intermediate zones requires applying denser FE meshes. Unfortunately, this makes the method impractical. Let us note that for the 4 × 4 mesh there exist 11,000 non-degenerated two-phase layouts in the cell Y characterized by the density ρ varying between (4/16, 16/16). For the 5 × 5 mesh, only for the selected value of ρ = 12/25 there exist (taking into account the symmetries of the cell) about 1,000,000 potential two-phase non-degenerated layouts. Consequently the computation time increases 100 times; for all the possible values of ρ, this time is increased 500 times. Note that the computation time of computing the homogenized moduli for the 4 × 4 mesh with using Xeon E3-1246 processor equals about 20 min, not taking into account possible optimization of the code. However, the selected patterns of the smallest approximation error (Equation (28)) may serve as the starting points for an inverse homogenization procedure. Let it be emphasized that the inverse homogenization is a non-convex and strongly non-linear problem due to the form of Equation (27). In this Equation, upon its relaxation involving the density ρ, all the entities are functions of the variable ρ referring to each element. A combinatorial search among the admissible microstructures (both isotropic and cubic) has made it possible to disclose the simple and fundamental auxetic microstructures. They can be served, as stated before, as the starting points to the further search of the extremal auxetic microstructures, i.e., such that they realize the Cherkaev–Gibiansky bounds for the 2D isotropy. In the case of cubic microstructures for which such bounds are not known, such microstructures would be patterns corresponding to the still unknown bounds.
